# Analysis of a cAMP regulated coactivator family reveals an alternative phosphorylation motif for AMPK family members

**DOI:** 10.1371/journal.pone.0173013

**Published:** 2017-02-24

**Authors:** Tim Sonntag, James J. Moresco, Joan M. Vaughan, Shigenobu Matsumura, John R. Yates, Marc Montminy

**Affiliations:** 1 Clayton Foundation Laboratories for Peptide Biology, The Salk Institute for Biological Studies, La Jolla, California, United States of America; 2 Department of Chemical Physiology, The Scripps Research Institute, La Jolla, California, United States of America; 3 Division of Food Science and Biotechnology, Graduate School of Agriculture, Kyoto University, Oiwake-cho, Kitashirakawa, Sakyo-ku, Kyoto, Japan; University of Louisville School of Medicine, UNITED STATES

## Abstract

The second messenger cAMP stimulates cellular gene expression via the PKA-mediated phosphorylation of the transcription factor CREB and through dephosphorylation of the cAMP-responsive transcriptional coactivators (CRTCs). Under basal conditions, CRTCs are phosphorylated by members of the AMPK family of Ser/Thr kinases and sequestered in the cytoplasm via a phosphorylation-dependent association with 14-3-3 proteins. Increases in cAMP promote the dephosphorylation and nuclear translocation of CRTCs, where they bind to CREB and stimulate relevant target genes. Although they share considerable sequence homology, members of the CRTC family exert non-overlapping effects on cellular gene expression through as yet unidentified mechanisms. Here we show that the three CRTCs exhibit distinct patterns of 14-3-3 binding at three conserved sites corresponding to S70, S171, and S275 (in CRTC2). S171 functions as the gatekeeper site for 14-3-3 binding; it acts cooperatively with S275 in stabilizing this interaction following its phosphorylation by the cAMP-responsive SIK and the cAMP-nonresponsive MARK kinases. Although S171 contains a consensus recognition site for phosphorylation by AMPK family members, S70 and S275 carry variant motifs (MNTGG**S**_**275**_LPDL), lacking basic residues that are otherwise critical for SIK/MARK recognition as well as 14-3-3 binding. Correspondingly, the activity of these motifs differs between CRTC family members. As the variant (SLPDL) motif is present and apparently phosphorylated in other mammalian proteins, our studies suggest that the regulation of cellular targets by AMPK family members is more extensive than previously appreciated.

## Introduction

The three members of the cyclic AMP (cAMP)-regulated transcriptional coactivator (CRTC) family share a common domain structure consisting of a conserved N-terminal cAMP response element-binding protein (CREB) binding domain (CBD), a central regulatory domain, and a C-terminal transactivation domain (TA) [1]. Both the CBD and the TA are required for CRTC transcriptional activity. The CBD mediates the interaction of CRTCs with the DNA-binding and dimerization domain (bZIP) of CREB family members (ATF1, CREB, and CREM), thereby facilitating the recruitment of these coactivators to relevant target genes [2]. Despite their apparent similarity, individual CRTCs exert non-overlapping effects on the expression of genetic programs in different tissues. CRTC2 and CRTC3 are co-expressed in hepatocytes, for example, but CRTC2 appears largely responsible in mediating effects of glucagon on hepatic gluconeogenic gene expression during fasting [3]. By contrast, CRTC3 appears critical in modulating effects of catecholamines on brown fat thermogenesis, while CRTC1 acts primarily in the CNS to control energy balance and entrainment of the circadian clock [4–6]. The molecular mechanism underlying the selective actions of individual family members is unclear, however.

Hormonal signals that stimulate intracellular cAMP concentrations increase gene expression via the phosphorylation of CREB and via the dephosphorylation of the CRTC family [1]. CREB phosphorylation at S133 induces a conformational change within the kinase-inducible domain (KID) that leads to the recruitment of the histone acetyltransferase coactivators CREB-binding protein (CBP) and p300 [7]. CRTC activity is primarily regulated by nucleo-cytoplasmic shuttling in response to phosphorylation and dephosphorylation at sites within the regulatory domain [8]. In the basal state, CRTCs are sequestered in the cytoplasm through phosphorylation at 14-3-3 binding sites by members of the AMPK family of Ser/Thr kinases, including the MARKs, SIKs, and AMPK itself [3, 8, 9]. Although they share sequence similarity and substrate recognition properties with AMPK, the SIKs and MARKs are not responsive to changes in cellular energy state [10]. Rather, MARKs have been implicated in cell polarity, while SIKs have been shown to regulate gene expression by modulating CRTC and class II HDAC nuclear localization [8, 11–13]. Increases in intracellular cAMP trigger the PKA-mediated phosphorylation and inhibition of the SIKs, but the relative roles of other AMPK family members in mediating effects of cAMP on cellular gene expression are not well understood [8, 14–16].

14-3-3 proteins bind as monomers or dimers to phosphorylated substrates that contain either MODE I (R[S/Ar][+/Ar]pS[L/E/A/M]P) or MODE II (Rx[Ar][+]pS[L/E/A/M]P; Ar = aromatic residue, + = basic residue, and x = any) recognition sites [17]. In keeping with the dimeric nature of 14-3-3s, most substrates harbor multiple 14-3-3 binding sites that are thought to act cooperatively in stabilizing this interaction [18]. The association with 14-3-3 proteins is also thought to induce conformational changes within the substrate [17–19], that may alternatively expose a nuclear export sequence or mask a nuclear localization sequence.

CRTCs contain three principal phosphorylation sites—(S70, S171, and S275 in CRTC2) that are regulated by cAMP and calcium signals, respectively–although the extent to which they and perhaps others modulate the activity of individual family members is unclear [8, 9, 20]. Here we evaluate the SIK and MARK dependent phosphorylation and 14-3-3 binding properties of each CRTC family member. We found that CRTCs contain alternative SIK/MARK phosphorylation motifs that may in turn contribute to the distinct regulatory properties of individual family members. These results may explain in part the non-overlapping role of these coactivators on cAMP-dependent transcription.

## Materials and methods

### Small molecules

Small molecules were solubilized in DMSO (ACS, Sigma-Aldrich) at the indicated concentrations and stored until usage at -80°C (long term storage) or -20°C (working dilution): 20 mM Forskolin (Sigma-Aldrich) and 2 mM Carfilzomib (PR-171) (Selleck Chemicals).

### Antibodies

The antibodies used in Western blot analysis were purchased from Santa Cruz Biotechnology (14-3-3 ε monoclonal & polyclonal), EMD Millipore (α-tubulin), Sigma-Aldrich (FLAG M2), Roche (Anti-HA-Peroxidase), Cell Signaling Technology‎ (14-3-3 [pan], CRTC1, P-CREB S133, P-CRTC1 S151, P-CRTC2 S171, P-MARK AL [AL = activation loop], P-PKA substrate), and Covance (GFP, HA.11). See Antiserum Production for the P-CRTC3 S273 antiserum (PBL #7378).

### Antiserum production

All animal procedures were approved by the Institutional Animal Care and Use Committee of the Salk Institute and were conducted in accordance with the PHS Policy on Humane Care and Use of Laboratory Animals (PHS Policy, 2015), the U.S. Government Principles for Utilization and Care of Vertebrate Animals Used in Testing, Research and Training, the NRC Guide for Care and Use of Laboratory Animals (8th edition) and the USDA Animal Welfare Act and Regulations. Three 10 to 12-week old, female New Zealand white rabbits, weighing 3.0 to 3.2 kg at beginning of the study, were procured from Irish Farms (I.F.P.S. Inc., Norco, California, USA). All animals were housed in an AAALAC accredited facility in a climate controlled environment (65–72 degrees Fahrenheit, 30–70% humidity) under 12-hour light:12-hour dark cycles. Rabbits were provided with ad libitum feed (5326 Lab Diet High Fiber), micro-filtered water and weekly fruits, vegetables and alfalfa hay for enrichment. Upon arrival, animals were physically examined by veterinary staff for good health and acclimated for two weeks prior to initiation of antiserum production. Each animal was monitored daily by the veterinary staff for signs of complications and weighed every two weeks. Routine physical exams were also performed by the veterinarian quarterly on all rabbits.

The rabbits were injected with a peptide fragment encoding Cys^268^pSer^273^ CRTC3(268–282) coupled to keyhole limpet hemocyanin via maleimide. The peptide, <Hnt>CNTGGpSLPDLTNLHY<NH2>, was synthesized and purified by Jean E. Rivier (The Salk Institute). The antigen was delivered to host animals using multiple intradermal injections of peptide-KLH conjugate in Complete Freund's Adjuvant (initial inoculation) or incomplete Freund's adjuvant (booster inoculations) every three weeks. Rabbits were bled, <10% total blood volume, one week following booster injections and bleeds screened for titer and specificity. Animals were administered 1–2 mg/kg Acepromazine IM prior to injections of antigen or blood withdrawal. At the termination of study, animals were exsanguinated under anesthesia (ketamine 50 mg/kg and aceprozamine 1 mg/kg, IM) and euthanized with an overdose of pentobarbital sodium and phenytoin sodium (1 ml/4.5 kg of body weight IC to effect). After blood was collected death was confirmed. All animal procedures were conducted by experienced veterinary technicians, under the supervision of Salk Institute veterinarians.

The antiserum obtained from the rabbit (code PBL #7378) with the best characteristics of titer and specificity was used for all experiments. So that the same batch of serum could be used for this and future studies, a large volume of serum from a single bleed was depleted of antibodies recognizing the non-phosphorylated form of CRTC by passing over a column containing Cys^268^CRTC3(268–282)-agarose resin. Covalent attachment of peptide to resin (Sulfolink coupling resin, Thermo Fisher) was per manufacturer's instructions.

### Plasmids

For overexpression studies we generated plasmids containing the *Homo sapiens* Ubiquitin C promoter (pUbC), whose activity is unaffected by cAMP signaling. pUbC was incorporated into three vectors (MCS = multiple cloning site): pUbC-3xFLAG-TEVsite-His6-MCS-IRESeGFP as well as pUbC-MCS-His6-TEVsite-3xFLAG-STOP-IRESeGFP (both pUbC-GFP backbone, addgene #11155), and pUbC-3xHA-MCS (pCHA backbone). Additional fragments integrated into the final vectors originated from the following sources: p3xFLAG-CMV™-14 (Sigma-Aldrich), pCeMM-CTAP(SG) [21], pTS48 [22], and pJL212 [23]. Remaining coding sequences and respective MCSs were introduced using primers, followed by multi-fragment ligation and inverse PCR to generate the final vectors.

The cDNAs (h = *H*. *sapiens*, m = *Mus musculus*) for the overexpression constructs originated either from existing lab plasmids (mCRTC1, mCRTC2, mCRTC3, mSIK2) or the Mammalian Gene Collection (MGC) / Harvard PlasmID Database (mNEK1, hMARK1, hMARK2, hMARK3, hMARK4, mMARK4, hMTFR1).

The plasmids used in this study were typically generated by restriction endonuclease based cloning, with final constructs coding for the following proteins (TV = transcript variant; in brackets UniProt identifier): mCRTC1 (Q68ED7-1), mCRTC2 (Q3U182-1), mCRTC3 (Q91X84-1), mNEK1(P51954-1), hMARK1 TV2 (Q9P0L2-1), hMARK2 TV3 (Q7KZI7-16), hMARK2 TV4 (Q7KZI7-1), hMARK3 TV3 (P27448-3), hMARK4 TV2 (Q96L34-2), mMARK4 (Q8CIP4-1), hMTFR1(Q15390-1), mSIK2 (Q8CFH6-1). All cDNAs derived plasmids, with the exception of hMTFR1, were N-terminally 3xFLAG-tagged; in case of mCRTC1-3 both N- and C-terminally tagged constructs were generated. In all experiments the N-terminal mCRTC1-3 plasmids were used, apart from IP-MS experiments were both the N- and C-terminally tagged CRTC proteins were subjected to the protocol.

Multi-step cloning was necessary for the following plasmids: mSIK2 (existing mutations in the ORF were reversed by site-directed mutagenesis to match Q8CFH6-1: T41I, R809Q) and hMARK2 TV4 (fusion PCR from two PCR products, template clones HsCD00331723 [TV3] and HsCD00323103 [incomplete TV4], assembled Q7KZI7-1).

All other mutations were generated via single or cumulative site-directed mutagenesis.

The luciferase reporter plasmid was generated by cloning the EVX promoter fragment (220 bp, 2x CRE half-sites; existing lab plasmid) into pGL4 (Promega).

Fusion PCR was performed as previously described [22].

### Cell culture

HEK293T cells were purchased from ATCC (CRL-11268) and propagated in DMEM media (Gibco®, high glucose) supplemented with 10% Fetal Bovine Serum (Gemini Bio-Products) and 100 U/ml penicillin-streptomycin (Corning Inc.).

### Overexpression & Immunoprecipitation (IP)

Experiments were performed in 6 well plates by reverse transfecting HEK293T cells (2.5 × 10^6^ cells) with 2 μg plasmid DNA using Lipofectamine® 2000 (Invitrogen). 48 h post transfection cells were collected in PBS and resuspended in lysis buffer (50 mM Tris, 150 mM NaCl, 10% glycerol, 1% Igepal [Sigma-Aldrich], 1 mM DTT, EDTA-free cOmplete™ Protease Inhibitor Cocktail [Roche], Phosphatase Inhibitor Cocktail 2 and 3 [Sigma-Aldrich], 1 μM Carfilzomib; pH 8.0). The supernatant (= cell lysate) was either used in IP experiments or directly mixed with SDS-PAGE loading buffer. In all IP experiments, cells were pre-treated for 1 h with 1 μM Carfilzomib prior to cell lysis. Cell lysate was incubated with anti-FLAG ® M2 magnetic beads and 3xFLAG peptide was used for elution (both Sigma-Aldrich).

### Immunoprecipitation & Mass Spectrometry (IP-MS)

IP-MS was performed in 6 x 100 mm dishes by reverse transfecting HEK293T cells (1.5 × 10^7^ cells) with 12 μg plasmid DNA using Lipofectamine® 2000 (Invitrogen). 48 h post transfection cells were treated for 1h with 1 μM Carfilzomib and 10 μM Forskolin. Cell collection and lysis was performed as described in the IP protocol.

Proteins were precipitated with 23% TCA and washed with acetone. Protein pellets were solubilized in 8 M urea, 100 mM Tris pH 8.5, reduced with 5 mM Tris(2-carboxyethyl)phosphine hydrochloride (Sigma-Aldrich), and alkylated with 55 mM 2-Chloroacetamide (Fluka Analytical). Proteins were digested for 18 h at 37°C in 2 M urea, 100 mM Tris pH 8.5, 1 mM CaCl_2_ with 2 ug trypsin (Promega). Five–step MudPIT analysis was performed using an Agilent 1200 G1311 quaternary pump and a Thermo LTQ Orbitrap Velos using an in-house built electrospray stage [24].

Protein and peptide identification and protein quantitation were done with Integrated Proteomics Pipeline—IP2 (Integrated Proteomics Applications). Tandem mass spectra were extracted from raw files using RawConverter [25] with monoisotopic peak option and were searched against a Uniprot *H*. *sapiens* protein database with reversed sequences and recombinant proteins added using ProLuCID [26, 27]. The search space included all fully-tryptic and half-tryptic peptide candidates with a fixed modification of 57.02146 on C and differential modification of 79.9663 on STY. Peptide candidates were filtered using DTASelect, with these parameters -p 2 -y 1—trypstat—pfp 0.01—modstat—extra—pI -DM 10—DB—dm -in -t 1—brief–quiet [25, 28]. Ascore was determined as in Beausoleil *et al*. [29].

### Immunofluorescence

HEK293T cells were plated in glass chamber slides (BD) and forward transfected with Lipofectamine® 2000 (Invitrogen) using mCRTC1 constructs in the pUbC-3xHA backbone. Roughly 24 h post-transfection cells were treated with either DMSO or 10 μM Forskolin for 30 min. Slides were fixed with 4% paraformaldehyde and incubated with primary antibodies (HA.11, CRTC1). Samples were incubated with secondary antibodies conjugated with Alexa Fluor® -488 (donkey anti-mouse) and -568 (donkey anti-rabbit) (Life Technologies) and subsequently counterstained with DAPI (Cayman Chemical Company) before images were acquired with a LSM 780 (Carl Zeiss).

### Luciferase reporter assays

Luciferase reporter assays were performed in 96 well plates by reverse transfecting HEK293T cells (100,000 cells). For each well 80 ng of DNA was used: 10 ng of EVX reporter plasmid, 10 ng of N-terminal FLAG-tagged CRTC1-3 plasmids, [20 ng of N-terminal FLAG-tagged kinase plasmid], 60 [40] ng of empty pUbC plasmid. 24 h post transfection Forskolin was added and cells further incubated for 4 h. DMSO served as the control treatment (each well 1% DMSO final). If cells were untreated, incubation occurred for 28 h post transfection. Next, 10 or 20 μl of Bright-Glo™ (Promega) was added per well and luciferase activity measured in a GloMax*®* multi microplate reader (Promega). All reporter assays were at least repeated twice and representative data is shown.

### Sequence alignment

Amino acid sequences were aligned using MegAlign and Clustal W method (DNASTAR v7).

## Results

### The phosphorylation status of conserved CRTC sites determines transcriptional activity

**Fig 1:**
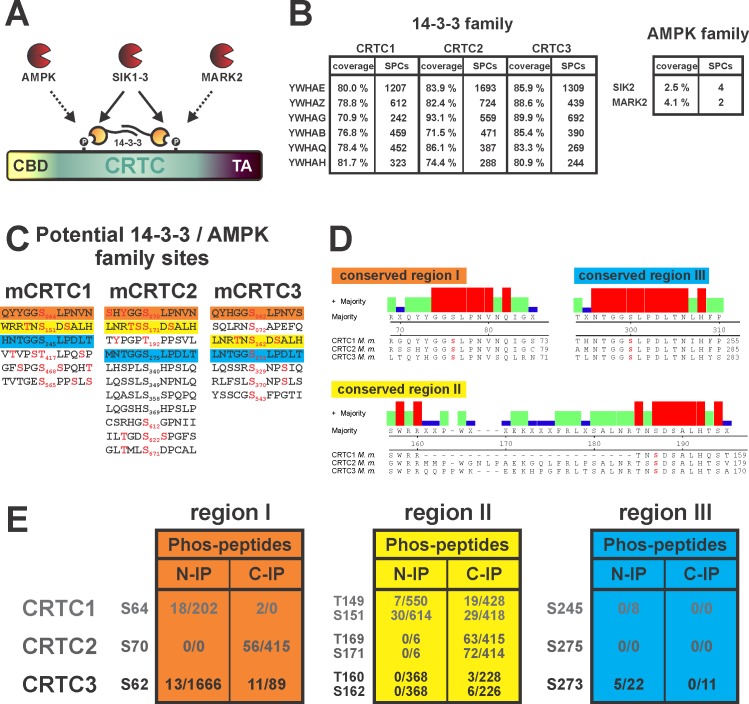
CRTC family members are phosphorylated at conserved sites that also mediate 14-3-3 binding. (A) Schematic representation depicting the inhibition of cAMP-regulated transcriptional coactivators (CRTCs) by AMPK family members (AMPK, SIK1-3, and MARK2). CRTC phosphorylation induces 14-3-3 binding and cytoplasmic sequestration. (CBD = CREB binding domain, TA = transactivation domain) (B) Table on the left shows the recovery of 14-3-3 protein family members (C-terminally tagged CRTCs) and table on the right recovered AMPK family members (arbitrary individual C-terminally tagged CRTC; SPCs = spectral counts). (C) List of the primary CRTC sequences of potential AMPK family phosphorylation sites (LXBS/TXSXXXL; B being a basic residue) that might simultaneously serve as 14-3-3 binding sites (RXXS/TXP). Amino acids shown in red were assigned to be phosphorylated by mass spectrometry. Three conserved CRTC regions are highlighted with boxes: orange (region I), yellow (region II), and blue (region III). (*Mus musculus* CRTC = mCRTC). (D) Sequence alignment of conserved regions I-III of *M*. *musculus* CRTC1-3. (E) Tables list the relative phosphorylation stoichiometry of key sites in conserved region I-III (spectral counts for phosphorylated versus non-phosphorylated). (B,C,E): N- and C-terminally FLAG-tagged CRTC1-3 (N-/C-IP) were overexpressed in HEK293T cells and stimulated with Forskolin prior to the IP-MS protocol.

In mass spectrometry (MS) studies to identify proteins that modulate CREB-dependent transcription, we recovered large quantities of 14-3-3 proteins from immunoprecipitates (IPs) of epitope-tagged CRTCs prepared from HEK293T cells, even following exposure to the adenylyl cyclase activator Forskolin (Fsk) (Fig 1A & 1B). In keeping with the proposed role of the AMPK family of Ser/Thr kinases in regulating CRTC activity, we also recovered MARK2 and SIK2 from CRTC IPs (Fig 1B). CRTCs contain a number of putative 14-3-3 binding sites that also correspond to canonical and variant phosphorylation motifs for AMPK members (Fig 1C). Three of these sites are conserved across the CRTC family (designated regions I, II, and III; Fig 1D) and are phosphorylated (S70, S171, S275; CRTC2 numbering) by MS analysis (Fig 1E). Each of these conserved serines have been reported as SIK phosphorylation sites [8, 20, 30].

**Fig 2:**
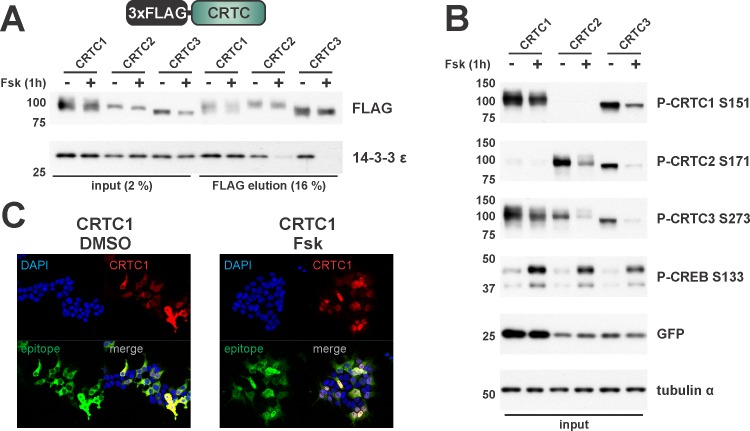
14-3-3 binding of CRTCs is modulated by cAMP. (A) and (B) Western blot analysis of the Co-IP of FLAG-tagged CRTC1-3. CRTCs were expressed from a plasmid containing the constitutive Ubiquitin C promoter (UbC), which drives the expression of EGFP via an internal ribosome entry site (IRES). In HEK293T cells the P-CREB S133 antibody recognizes two bands, the upper band being CREB1 and the lower band ATF1 (calculated molecular weight: ~ 37 and 29 kDa). (DMSO/Fsk treatment for 1h) (C) Immunofluorescence of HEK293T cells transfected with HA-tagged CRTC1. Cells were co-stained for HA & CRTC1 and counterstained with DAPI. (DMSO/Fsk treatment for 30 min)

Binding of CRTCs to 14-3-3s sequesters them in the cytoplasm; and increases in cAMP promote the dephosphorylation and release of CRTCs from 14-3-3 proteins leading to their nuclear localization [8, 20, 31]. Exposure of HEK293T cells to Fsk reduced 14-3-3 protein binding to CRTC2 and CRTC3 (Fig 2A), coinciding with dephosphorylation of regions II/III (S171 and S275, CRTC2 numbering) (Fig 2B) and their translocation to the nucleus (data not shown). By contrast, Fsk treatment had only modest effects on the dephosphorylation of CRTC1 at regions II and III (S151 and S245) or on its liberation from 14-3-3 proteins and nuclear translocation (Fig 2A–2C). These results indicate that CRTC1 is activated more weakly in response to cAMP signals compared to CRTC2 and CRTC3, confirming previous reports [31].

**Fig 3:**
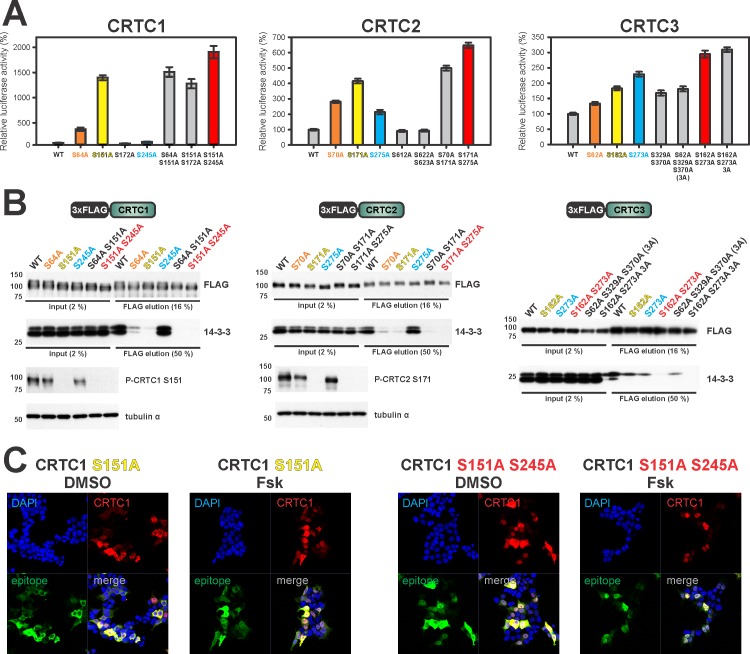
Characterization of 14-3-3 binding sites in the CRTC family. (A) EVX-Luc reporter assay of putative 14-3-3 site mutants inside CRTC1-3. (n = 10, ±s.e.m.) (B) Western blot analysis of the Co-IP of FLAG-tagged CRTC 14-3-3 mutants. (C) Immunofluorescence of HEK293T cells transfected with HA-tagged CRTC1 14-3-3 mutants. Cells were co-stained for HA & CRTC1 and counterstained with DAPI. (DMSO/Fsk treatment for 30 min). (A-C): Ser/Ala mutations of the three conserved CRTC sites are highlighted (region I = orange, region II = yellow, blue = region III, and double mutant of site II and III in red)

We performed Ser/Ala mutagenesis of individual phospho-acceptor sites within each conserved region to determine their effects on CRTC transcriptional activity by transient assay with a luciferase reporter containing cAMP responsive elements (CREs) [32]. For each family member, mutation of site II substantially upregulated CRE reporter activity relative to wild type (CRTC1 S151A ~15-fold, CRTC2 S171A ~4-fold, and CRTC3 S162A ~2-fold), whereas Ser/Ala mutations in regions I and III had more variable effects (Fig 3A). Mutation of region I strongly induced CRTC1 activity (S64A ~4-fold), for example, but it had only modest effects on CRTC3 (S62A). Conversely, region III strongly induced CRTC3 activity (S273A ~2-fold), but not CRTC1. 14-3-3 proteins can bind phosphorylated substrates as dimers. Indeed, a number of 14-3-3 target proteins contain multiple binding sites that act cooperatively to stabilize this interaction [33]. Consistent with this observation, mutation of both S171 and S275 substantially increased the activity of all family members, but combined mutations at other sites did not (Fig 3A). Having seen effects of mutations at the conserved phospho-acceptor sites on transcriptional activity of individual CRTCs, we examined whether these mutations also modulate 14-3-3 binding. CRTC mutations in site II always reduced 14-3-3 binding, but mutations in site I and III had differential effects (Fig 3B). For example, mutation of the phospho-acceptor site in region III did not appear to modulate the binding of CRTC1 and CRTC2 to 14-3-3s, but it readily reduced 14-3-3 binding in the context of CRTC3. Nevertheless, the cooperativity between sites II and III we observed in CRE reporter assays was also evident in 14-3-3 binding and in nuclear localization studies (Fig 3B & 3C).

### Primary structure of region II modulates CRTC activity and 14-3-3 binding

**Fig 4:**
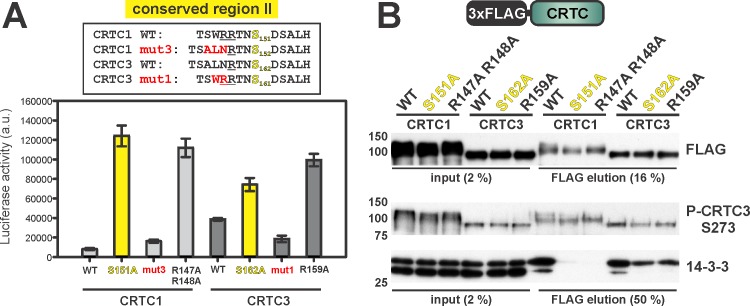
Arginine residues inside a SIK/MARK phosphorylation site regulate 14-3-3 binding and transcriptional activity of CRTCs. (A) EVX-Luc reporter assay comparing arginine mutants inside region II of CRTC1 and CRTC3. Phosphorylated region II serines (yellow), arginine residues (underlined), and mutated amino acids (red) are highlighted. (n = 10, ±s.e.m.) (B) Western blot analysis of the Co-IP of corresponding FLAG-tagged arginine CRTC1 and CRTC3 mutants.

Having seen that mutation of region II in CRTC1 (S151A) has a stronger effect on 14-3-3 binding and CRE reporter activity compared to the corresponding mutant in CRTC3 (S162A), we examined the potential role of residues flanking this phospho-acceptor site in modulating the transcriptional activity of individual family members. S151 in CRTC1 is preceded by two arginine residues at positions (-4, -3), whereas CRTC2 and CRTC3 contain only a single arginine (Fig 1D). Because arginine residues contribute substantially to AMPK family-mediated phosphorylation and to 14-3-3 binding affinity [10, 33, 34], we examined whether these residues can account for the differences in cAMP responsiveness of CRTC1 compared to CRTC2 and CRTC3. For both CRTC1 and CRTC3, mutation of the arginine residues in site II to alanine decreased 14-3-3 binding, leading to increases in CRE reporter activity that were comparable to effects of phospho-acceptor serine mutations (S151A and S162A, Fig 4A & 4B). Exchange of three residues N-terminal to phospho-acceptor site II in CRTC1 (W_146_RR) and CRTC3 (L_157_NR) (CRTC1 mut3 and CRTC3 mut1, respectively) decreased CRTC3 activity more than 50% and increased CRTC1 more modestly (Fig 4A). These results suggest that increases in 14-3-3 binding affinity at site II contribute to the weak induction of CRTC1 by cAMP.

### AMPK family members repress CRTCs with distinctive patterns

**Fig 5:**
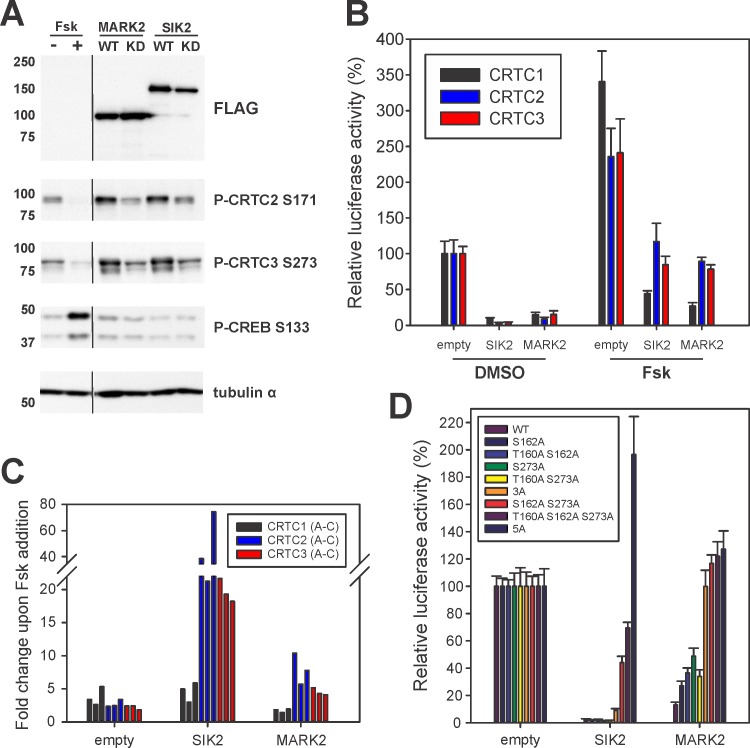
Multi-site phosphorylation of CRTCs by SIK and MARK kinases. (A) Western blot analysis of the overexpression of FLAG-tagged wild type (WT) versus kinase dead (KD) SIK2 and MARK2 (SIK2 KD = T175A, MARK2 KD = T208A S212A). The P-CRTC3 S273 antibody recognizes two bands in HEK293T cells, the upper band being CRTC2 and the lower band CRTC3 (calculated molecular weight: ~ 73 and 67 kDa). (DMSO/Fsk treatment for 1h) (B) EVX-Luc reporter assay comparing the effect of AMPK family members SIK2 and MARK2 co-expression on the transcriptional activity of CRTC1-3. EVX-Luc activity was measured after 4 h of DMSO or Fsk treatment. (n = 5, ±s.e.m.) (C) Graph depicting the corresponding fold changes in reporter activity upon Fsk treatment in three independent experiments (A-C, A = results depicted in Fig 4B). (D) EVX-Luc reporter assay comparing the effect of SIK2 and MARK2 co-expression on the transcriptional activity of CRTC3 14-3-3 mutants (3A = S62A S329A S370A, 5A = S62A S162A S273A S329A S370A). (n = 10, ±s.e.m.)

Although members of the AMPK family have been shown to modulate CREB target gene expression through inhibitory phosphorylation of the CRTCs, their relative activities have not been examined [3, 8, 9]. Based on the recovery of MARK2 and SIK2 but not other AMPK family members from IPs of individual CRTCs, we examined effects of these kinases on CREB activity. Overexpression of wild type but not catalytically inactive SIK2 or MARK2 increased amounts of phosphorylated endogenous CRTC2/3 at conserved sites II and III by Western blot analysis with phospho-specific antisera directed against S171 and S275 (in CRTC2) (Fig 5A). SIK2 or MARK2 also decreased basal CRE reporter activity in cells co-expressing individual CRTC family members (SIK2 ~5% and MARK2 ~20% of wild type CRTC activity; Fig 5B).

cAMP has been reported to inhibit SIKs and other AMPK family members through PKA-mediated phosphorylation [1, 35]. Indeed, exposure to Fsk decreased endogenous CRTC2 and CRTC3 phosphorylation at sites II and III (Fig 5A). Similarly, Fsk treatment increased CRTC2 and CRTC3 activity in CRE reporter assays of HEK293T cells co-expressing SIK2 and, to a lesser extent, MARK2 (Fig 5B & 5C). Fsk had more modest effects on CRTC1 activity in cells overexpressing SIK2 or MARK2, likely reflecting a requirement for dephosphorylation by the Ca^2+^-dependent Ser/Thr phosphatase calcineurin [31, 36].

Knowing that AMPK family members inhibit CRTCs primarily through phosphorylation at sites II and III, we reasoned that mutant CRTC proteins containing alanine substitutions at these sites should be resistant to repressive effects of SIKs and MARKs. Although it disrupted the activities of individual site II and III CRTC3 mutants, SIK2 over-expression had only modest effects on site II/III double mutants and no effect on the activity of a mutant with alanine substitutions at both conserved and non-conserved sites (WT ~3%, S162A S273A ~50%, and 5A = S62A S162A S273A S329A S370A ~200% activity) (Fig 5D). Consistent with its lower potency for CRTCs, MARK2 co-expression had minor effects on the activities of CRTC3 phospho-mutants. These results indicate that phosphorylation sites unique to individual CRTC family members likely contribute to their distinct profiles of transcriptional activation.

#### Regulation of CRTCs by MARK kinase family members

**Fig 6:**
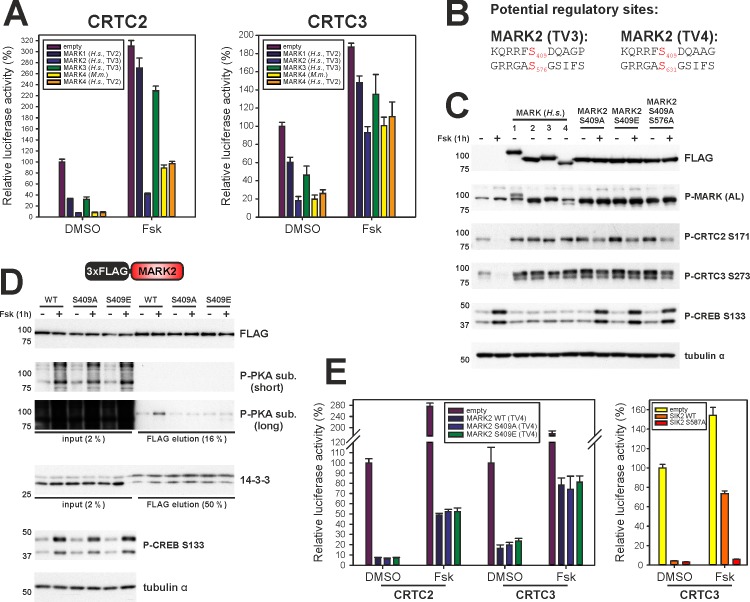
Effects of MARK family members on CRTC phosphorylation and cAMP dependent gene expression. (A) EVX-Luc reporter assay comparing the effect of MARK kinase family co-expression on the transcriptional activity of CRTC2 and CRTC3 (TV = transcript variant). EVX-Luc activity was measured after 4 h of DMSO/Fsk treatment. (n = 5, s.e.m.) (B) Primary sequences of potential PKA sites in *H*. *sapiens* MARK2 (TV3 = 1–724; TV4 1–788). Phosphorylated residues are shown in red. (C) Western blot analysis of the overexpression of FLAG-tagged MARK kinase family members and MARK2 (TV3) mutants. (P-MARK (AL) = P-MARK activation loop, DMSO/Fsk treatment for 1h) (D) Western blot analysis of the Co-IP of FLAG-tagged MARK2 (TV3) mutants. (DMSO/Fsk treatment for 1h) (E) EVX-Luc reporter assay measuring the effect of MARK2 S409 (TV4) mutants upon the transcriptional activity of co-expressed CRTC2 and CRTC3. As a control, a EVX-Luc reporter assay with SIK2 and known PKA-site mutant (S587A) with co-expressed CRTC3 was performed. EVX-Luc activity was measured after 4 h of DMSO/Fsk treatment. (n = 5, ±s.e.m.)

Based on the ability of MARK2 to repress CRTC activity by multi-site phosphorylation, we examined the roles of other MARK family members in this process. Each family member was competent to inhibit CRE reporter activity, with MARK2 and MARK4 appeared to be most potent. Overexpression of MARK 2/4 inhibited the activities of both CRTC2 and to a lesser extent CRTC3 (Fig 6A). Despite the presence of a consensus PKA phosphorylation site at S409 (Fig 6B), which has been reported to modulate MARK2 activity [35], phospho-mimetic and phospho-incompetent mutations at S409 had no effect on MARK2-mediated phosphorylation of CRTC2/3 at sites II and III (Fig 6C). We confirmed that S409 is indeed phosphorylated in response to Fsk stimulation by using a phospho-PKA substrate antibody (Fig 6D). Correspondingly, MARK2 mutant proteins containing Ala or Glu substitutions at S409 were not phosphorylated in response to FSK. However, neither S409 mutants nor Fsk had effects on MARK2’s ability to bind 14-3-3 proteins (Fig 6D) or MARK2-mediated inhibition in the CRE reporter assay (Fig 6E). By contrast, mutation of a conserved PKA phosphorylation site in SIK2 (S587A) [8, 14], disrupts inhibitory effects of FSK on SIK2 activity, demonstrating that cAMP selectively modulates SIK but not MARK activity towards the CRTCs.

### Phosphorylation of the site III variant motif by SIKs and MARKs

**Fig 7:**
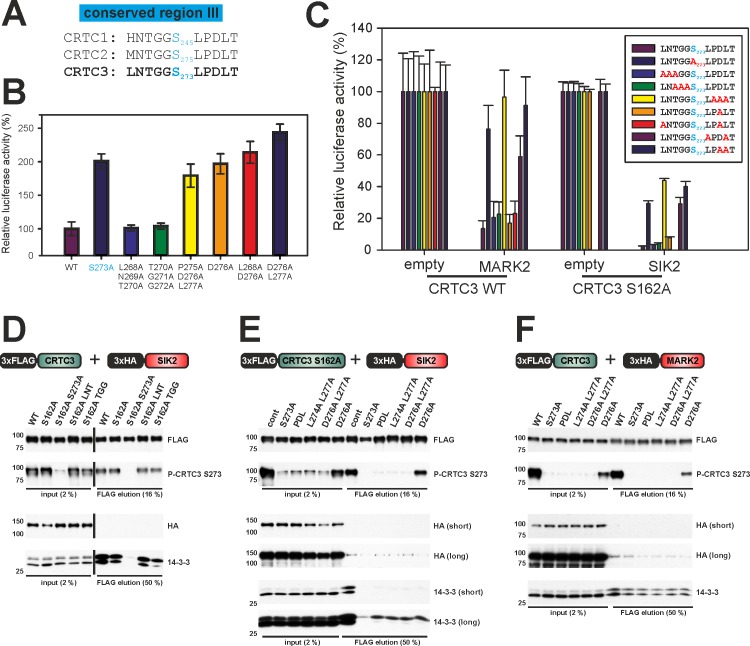
A variant SIK2/MARK2 recognition site modulates CRTC activity. (A) Sequence alignment of CRTC conserved region III. The phosphorylated region III serines are shown in blue. (B) EVX-Luc reporter assay comparing the transcriptional activity of CRTC3 carrying mutations in region III. (n = 10, ±s.e.m.) (C) EVX-Luc reporter assay measuring the effect of SIK2 or MARK2 co-expression on the transcriptional activity of CRTC3 region III mutants (red). In case of SIK2 the mutants were assayed in the background of the S162A mutant. (n = 5, ±s.e.m.) (D) & (E) Western blot analysis of the Co-IP of FLAG-tagged CRTC3 region III mutants upon co-expression of HA-tagged SIK2. F) Western blot analysis of the Co-IP of FLAG-tagged CRTC3 mutants upon co-expression of HA-tagged MARK2. (cont = control / CRTC3 S162A, LNT = L268A N269A T270A, TGG = T270A G271A G272A, PDL = P275A D276A L277A)

Although SIKs and MARKs appear to phosphorylate CRTCs comparably at regions II and III, region III lacks a basic residue within the LXBS/TXSXXXL (B = basic amino acid) motif [3, 8], which appears critical for phosphorylation by these basophilic kinases (Fig 7A). Moreover, by contrast to the disruptive effects of mutations N-terminal to the phospho-acceptor site in region II (Fig 4), alanine mutagenesis of similar residues in region III (S273), most notably L268—a key hydrophobic residue at -5 within the consensus recognition motif (L_268_NTGG)—had no discernable effect on CRTC3 activity (Fig 7B). Yet, mutation of CRTC3 at residues C-terminal to S273 (L_274_PDL), increased its transcriptional activity, indicating that these sites are required for inhibitory phosphorylation and/or 14-3-3 binding. Correspondingly, CRTC3 proteins containing two mutations in the L_274_PDL sequence were more resistant to inhibitory effects of MARK2 or SIK2 over-expression on CRE reporter activity (Fig 7C). Consistent with their effects in reporter assays, CRTC3 L_274_PDL mutants also had reduced binding affinity for 14-3-3s and for SIK2/MARK2 (Fig 7D–7F). Notably, the sequences flanking site I (GGSLPNV) are conserved to a substantial extent in region III (GGSLPDL), possibly indicating that these sites are differentially regulated relative to site II.

## Discussion

**Fig 8:**
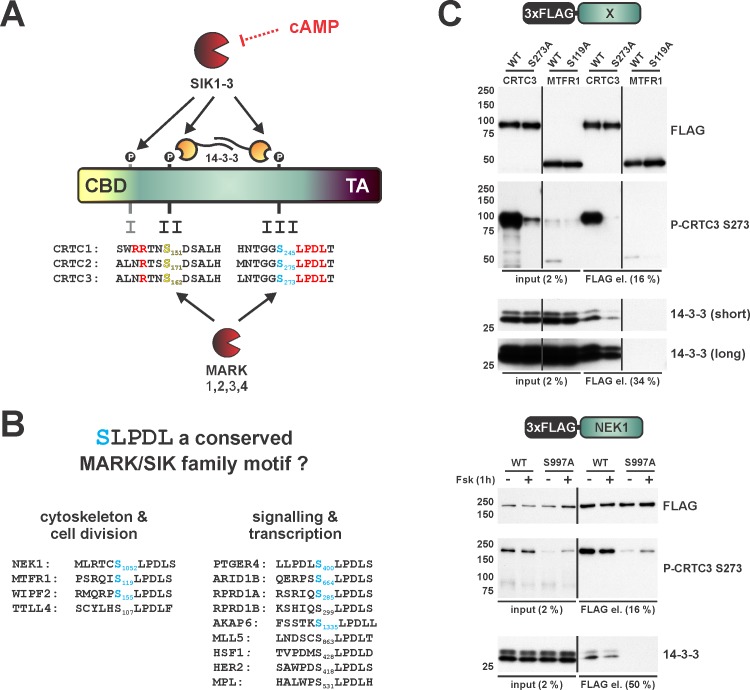
Role of the SLPDL motif in the SIK and MARK kinase-mediated phosphorylation of cellular substrates? (A) Schematic representation depicting the CRTC activation/inactivation mechanism. SIK and MARK mediated phosphorylation at multiple conserved sites leads to 14-3-3 binding and cytoplasmic sequestration. Highlighted amino acids are either phosphorylated 14-3-3 sites II (yellow) and III (blue) or essential for phosphorylation and 14-3-3 functionality (red). The second messenger cAMP strongly inhibits SIKs via PKA-mediated phosphorylation, while MARKs CRTC directed activity remains unaffected. (B) Tables list *H*. *sapiens* proteins harboring the SLPDL motif. Serines shown in blue were found to be phosphorylated in the PhosphoSitePlus database [37]. (C) Western blot analysis of the Co-IP of FLAG-tagged CRTC3, MTRF1 (*H*. *sapiens*, isoform 1), and NEK1 (*M*. *musculus*). Correspondingly, wild type (WT) and SLPDL motif phospho-acceptor site mutants (mCRTC3 S273A, hMTFR1 S119A, and mNEK1 S997A) were compared with each other (NEK1: DMSO/Fsk treatment for 1h).

cAMP has been shown to stimulate CREB target gene expression through the dephosphorylation of CRTCs at sites that otherwise mediate 14-3-3 protein binding and cytoplasmic sequestration (Fig 8A). We found that SIKs and MARKs associate with and regulate CRTC activity. MARK2 and MARK4 are the most potent members of the MARK family, although they are less effective than SIK2 in regulating CREB target gene expression. Moreover, MARK activity towards CRTCs is not modulated by cAMP signaling, demonstrating that SIKs are the likely physiologic mediators of cAMP dependent transcription.

Members of the AMPK family are basophilic kinases that phosphorylate substrates containing LXBS/TXSXXXL (B = basic amino acid) motifs. These kinases often phosphorylate substrates at 14-3-3 binding sites, which also contain arginine residues at position -3/-4 relative to the phospho-acceptor serine [38]. Of the three conserved phosphorylation sites in the CRTCs, only region II contains a consensus site with basic residues at -3 or -4. We found that arginine residues N-terminal to region II are critical for binding of CRTCs to 14-3-3, because Arg/Ala substitutions reduced 14-3-3 binding comparably to phospho-acceptor site mutations. Indeed, the presence of two Arg residues at -3 and -4 in CRTC1 actually enhances its affinity for 14-3-3 proteins and is likely responsible for its weak induction by cAMP relative to CRTC2 and CRTC3. In contrast to region II, regions I and III contain variants that are nevertheless phosphorylated by SIKs and MARKs. Both sites lack Arg residues, hence these variants would appear to have lower affinity for 14-3-3s individually. However, because of the dimeric nature of 14-3-3 proteins, these variants function cooperatively with the gatekeeper site in region II.

The key functional feature of region III is the SLPDL motif, which resembles the inhibitory repeat motif (K_955_VDDL) in the *Helicobacter pylori* virulence factor CagA, a known MARK2 peptide mimetic [39]. Using an *in silico* search for mammalian proteins that carry the SLPDL motif, we identified multiple potential targets in pathways where MARKs and SIKs may perform key regulatory roles (Fig 8B) [40]. Indeed, several of these putative SIK/MARK recognition sites appear to undergo phosphorylation, according to the curated PhosphoSite Plus database [37]. NIMA(never in mitosis gene a)-related kinase 1 (NEK1) and mitochondrial fission regulator 1 (MTFR1) proteins appear to be robustly phosphorylated at the SLPDL motif [37]. Correspondingly, over-expressed MTFR1 and NEK1 are recognized by the CRTC3 S273 phospho-specific antiserum, but phosphorylation-defective MTFR1 (S119A) and NEK1 (S997A) mutants are not (Fig 8C). Similar to the CRTCs, NEK1 appears to interact with 14-3-3 proteins via the phosphorylated SLPDL motif. NEK1 has been shown to translocate from the cytoplasm to the nucleus upon DNA damage, where it is thought to modulate homologous recombination [41, 42]. Notably, mutations in NEK1 have been associated with ciliopathy (short-rib thoracic dysplasia 6 with or without polydactyly [SRTD6], homozygous mutation) and amyotrophic lateral sclerosis (ALS, heterozygous mutation) [43, 44], Our data suggests that the non-canonical SLPDL motif corresponds to a distinct site for phosphorylation by AMPK family members as well as 14-3-3 protein binding. Future studies on NEK1 and other substrates should provide insight into this process.
